# Impact of COVID-19 on the education and healthcare services of persons with disabilities in Lebanon

**DOI:** 10.3389/fpubh.2023.1111972

**Published:** 2023-06-19

**Authors:** Itab Shuayb, Sabine Doueiry

**Affiliations:** Centre for Lebanese Studies, Cambridge University, Beirut, Lebanon

**Keywords:** COVID-19, Lebanon, healthcare, education, persons with disabilities (PWDs)

## Abstract

This study examines the impact of the compounding crises that engulfed Lebanon during the COVID-19 pandemic on the schooling and healthcare opportunities and experiences of persons with disabilities. It further explores how disability interacts with other forms of discrimination, such as gender and socioeconomic factors, exacerbating the risk of exclusion from mainstream education and healthcare services. Qualitative research methods were employed to delve into the complexities of these issues. The researchers conducted a comprehensive review of 37 relevant COVID-19 reports, research studies, guidelines, documents, and rapid analysis studies generated by the Lebanese Ministry of Public Health, local and international NGOs, and UN agencies. Additionally, analysis of social media content and COVID-19 awareness campaigns was carried out to assess their accessibility and acknowledgment of the needs of persons with disabilities (PWD). Furthermore, eighteen virtual open-ended interviews were conducted with adults with disabilities, parents of children with disabilities, local and international disability organizations, and representatives from the education and healthcare sectors. The findings from the interviews revealed that while the COVID-19 pandemic disrupted the daily routines of individuals, persons with disabilities encountered additional barriers on top of the pre-existing ones they faced before the implementation of lockdown restrictions. The study identified five overarching themes at the policy and decision levels, as well as the academic and healthcare services levels, which hindered persons with disabilities from accessing education and healthcare. Drawing from the five main themes, this study presents and discusses key findings, implications, and recommendations. These findings shed light on the challenges faced by persons with disabilities during the compounding crises and the implications for their access to education and healthcare. The study provides recommendations to address these issues and improve the opportunities and experiences of persons with disabilities in times of crisis.

## Introduction

In Lebanon, children are facing a “lost year” of learning amidst the overlapping crises that have put the education system at risk of collapse (1). The October 17, 2019, uprising, the COVID-19 pandemic, the Beirut explosion of August 4, 2020, the ongoing financial crisis, the currency devaluation, and the nationwide electricity crisis have added more educational challenges, particularly for low-income families and persons with disabilities (PWD). Learning losses have also been proportional to existing educational inequalities further exacerbated by pandemic restrictions. Communities already disadvantaged and excluded from adequate resources before the pandemic faced more severe consequences (2, 3). Those include children from low-income households, children with disabilities, and girls who had limited availability of electricity, connectivity, devices, accessible technologies, and early intervention services and faced discrimination and social/gender norms. Other student groups facing more learning losses than others are younger students with less access to age-appropriate remote learning and pre-school-age students who were often excluded from remote education (4–7). Students whose first language is not English also faced more learning losses than others (8).

Prior to the COVID-19 pandemic, the education system in Lebanon was characterized by limited accessibility for persons with disabilities. Despite the provision of equal educational opportunities for persons with disabilities in Law 220/2000, inclusive education has not been effectively promoted in schools and universities due to the existence of special care educational institutions and special schools (9,10). Article 59 of the law provides for the right to education for persons with disabilities, including access to both regular and special classes, while Article 60 prohibits discriminatory barriers for PWDs when seeking admission to educational establishments, whether public or private (10, 11).

Inclusive education is a vital component of ensuring equal opportunities and social inclusion for individuals with disabilities (10, 12, 13). However, many students with disabilities in Lebanon are excluded from mainstream schools due to a lack of reasonable accommodations, including inclusive policies and curricula, trained staff, adapted equipment, financial support, and accurate data on children with disabilities (14, 15).

The economic crisis that has plagued Lebanon since 2019 has further exacerbated the challenges faced by students with disabilities. Limited resources, power cuts, slow internet connections, unsuitable learning environments at home, high costs of educational devices, and a lack of education among parents and caregivers have all contributed to the restricted access to online education for students with disabilities (16, 17).

During the October 17, 2019, uprising, many public and private schools in Lebanon had to close due to road closures. Many private schools offered online education during road closures; however, most public schools and some private schools were less prepared and had to close their schools. Since the beginning of the pandemic, schools in Lebanon have closed for a total of 49 weeks beginning March 2, 2022 (18). The Ministry of Education announced that all public and private schools, special education centres and universities must close by February 29, 2020. Over 1.3 million children have been affected, of which over 700,000 dropped out of school due to financial difficulties (19). In addition, the UN estimates that 100,000 to 120,000 children were transferred to public schools between 2019 and 2021 due to the inability to cover school tuition (1). Some families even chose to give priority to educating their children without disabilities.

According to Human Rights Watch (20), the Lebanese Ministry of Education and Higher Education (MEHE) ordered schools to close during the Covid pandemic. Nevertheless, it did not have a clear plan to amend the curriculum contents and objectives to be aligned with the online educational model requirements. Furthermore, the Ministry of Education did not provide reasonable accommodations or laptops/tablet devices for parents of students with disabilities who lacked the financial resources to purchase such educational equipment. To compensate for the ministry’s support, some local disability organizations provided financial support and distributed tablet devices for low-income families with children with disabilities. In contrast, other local and international NGOs amended their educational curriculum to be delivered online (20).

Some private schools shifted their education to online learning during the lockdown restrictions as they relied on online learning during October 17, 2019, road closures. Other schools could not move immediately to remote schooling as they had to review and adjust their curriculums for online education (21). Therefore, teaching and learning methods were often inaccessible and did not accommodate the needs of students with disabilities during the COVID-19 pandemic. In fact, with insufficient access to inclusive distance learning tools, students have not received proper education for almost two academic years (22).

On the other hand, the Beirut Port Blast caused much infrastructural damage to many schools and hospitals in Beirut, which had to close their premises for reconstruction purposes. Furthermore, many schools and special education institutions were severely affected by the economic crisis and could not provide inclusive educational support for their students with disabilities during remote learning. Other special education centres relied on the government’s funding to support students with disabilities; however, the government’s reimbursement payment delays forced many teachers and special educators. Leaving these centres and seeking stable and better job opportunities affected the quality of educational services at these special academic centres. Acknowledging all of these constraints, several international disability organizations provided educational support for students with and without disabilities. Hence UNICEF, the United Nations Educational, Scientific and Cultural Organization (UNESCO), and Food and Agriculture Organization (FAO) provide educational and financial support and food supplements for students with disabilities to pursue their online learning (20).

Since the beginning of the pandemic, schools in Lebanon have closed for 49 weeks (18). On February 29 2020, most schools and universities in Lebanon had to close, including special education schools and centres. According to Human Rights Watch (20), the Lebanese Ministry of Education had ordered schools to close; however, it neither provided clear guidance on continuing educational curriculum remotely nor provided accommodations or educational, technological devices for families who could not afford to buy tablets and computer devices. Without clear guidance from the ministry of education, many special educational institutions and disability local and international non-governmental organizations took the lead and initiative to tailor their curriculum and deliver their teaching methods remotely (20).

The pandemic and the economic crisis happening simultaneously hindered school reopening in Lebanon. According to UNESCO (18), despite the Omicron variant, schools in Lebanon are open; however, schools were struggling to afford essential items, including stationery, computer equipment, hygiene material, and teachers’ income, all while functioning on a few hours of electricity per day. In September 2022, many private schools managed to provide the necessary resources; hence, they could reopen their doors. Other private schools increased tuition by as much as 80 percent and asked for payments in US dollars instead of Lebanese pounds (1). On the other hand, public schools postponed their opening and launched an open-ended strike to demand higher wages. Teachers’ salaries were not adjusted to reflect the Lebanese currency’s 90 percent devaluation coupled with the surging inflation rate (23). As a result, teachers struggled to meet their teaching objectives and expressed having to complete unpaid work in a study by the Centre for Lebanese Studies. Students attributed teachers’ struggles to effectively teach online to the regression of the quality of learning, whereby they are not ready to sit for official exams this year (19). Moreover, some families of children with disabilities had difficulty helping their children as they did not know about specific courses (24, 25).

According to Human Rights Watch (1), Lebanon is obliged under human rights laws to provide free primary education even during economic crises and pandemics. However, despite the difficulties mentioned above happening over 2 years, the Lebanese government failed to properly plan for funding educational needs or guide schools, teachers, and parents to compensate for the education loss.

### COVID-19 and the healthcare services for vulnerable populations

During the economic crisis and the COVID-19 pandemic, both the educational and healthcare services in Lebanon did not anticipate the needs of persons with disabilities, low-income families, children, teenagers, the elderly, migrants and refugees who are among the vulnerable populations.

In Lebanon, the Ministry of Social Affairs (MOSA) estimates the number of persons with disabilities as 80,703 persons (26), while the UNESCO (18) report mentions that around 15% of the total population in Lebanon has disabilities.

The Ministry of Public Health (MOPH) implemented measures on February 22, 2020, to track the spread of the COVID-19 virus. The procedures included testing the suspected cases with COVID-19 symptoms, holding the positive cases in quarantine centres and hospitals, and requesting the local municipalities and the community to enforce home isolation and quarantine for those with asymptomatic conditions. Furthermore, the Lebanese Ministry of Health coordinated with municipalities to physically trace the inspected cases since it lacked the technological tracing mechanisms and logistics. While these procedures aimed to limit the spread of the COVID-19 virus, they created barriers and restricted persons with disabilities from accessing healthcare services during the lockdown restrictions (20). The Ministry of Health neither consulted nor involved persons with disabilities and local and international disability organizations during its emergency response preparation. Hence most of the Ministry of Health’s COVID-19 information was inaccessible to persons with disabilities.

Even though Law 220/2000 prohibits discrimination against persons with disabilities (PWDs), many PWDs continue to face significant barriers in the built environment and access to mainstream education, employment and healthcare services. The lack of accessible services before and during the COVID-19 pandemic has forced many persons with disabilities to request medical assistance from local and international disability non-governmental organizations (NGOs and INGOs) during the lockdown restrictions. Many local and international NGOs had to distribute medications, personal hygiene kits, and respirators for persons with disabilities who had chronic health illnesses (20). The Lebanese Ministry of Health launched several COVID-19 awareness advocacy campaigns to prevent the spread of the virus just 4 days after the first document COVID-19 infected case (27). However, most media content was not accessible to persons with disabilities. To fill that gap, UNICEF, the Disability Hub within the Centre for Lebanese Studies at the Lebanese American University, and the Lebanese Federation of the Deaf produced several COVID-19 short video awareness campaigns and written information materials accessible for people with mobility, sensory and cognitive disabilities. According to (28), people with visual disabilities rely on touching objects, and many of them use different auxiliary aids and mobility devices that can increase the spread of the virus if they are not adequately washed or cleaned. Hence, it is vital to provide accessible COVID-19 hygiene and prevention information to enable persons with disabilities to follow the hygiene procedures to limit the spread of the virus among them and their family members. Although the (29) produced many COVID-19 hygiene guidelines and procedures to disinfect hands and surrounding surfaces regularly, there were few guidelines for persons with disabilities about ways to properly disinfect their aids (30).

Moreover, many persons with disabilities with low incomes could not have enough money to obtain cleaning products (31). According to (28), persons with low vision rely on touching surfaces to navigate around their surroundings, increasing their chances of being infected by the Covid-19 virus. In contrast, people with multiple disabilities and those who rely on caregivers cannot maintain the required 1.5-meter social distance (16).

On the other hand, the Lebanese Ministry of Public Health (MOPH) presented several media conferences to inform the community about the COVID-19 situation; however, none of these conferences was accompanied by Lebanese sign language interpreters. According to the Arab Organization of Persons with Disabilities (32), several people from the Deaf community rely on sign language interpretation to get updates and news about their community. However, many people who use hearing aids rely on captions and subtitles to gain access to audible information, as not many of them understand sign language (30, 33). Many persons with disabilities or their caregivers infected with the COVID-19 virus were frequently moved to hospitals and quarantine centres and were isolated from their caregivers and family members. Such isolation hindered many persons with disabilities from receiving the care they needed, while people with cognitive and hearing disabilities found difficulties communicating with the medical team and staff (34, 36). On the other hand, many persons with disabilities could not take the PCR tests, and others could not access hospitals and testing centres in Lebanon as many of them were inaccessible for persons with disabilities.

The Lebanese Ministry of Public Health never documented the number of persons with disabilities who have contracted the virus nor those who died after being infected. However, its official statistics included information about the number of infected cases without disabilities, death tolls, and affected regions (17).

## The current study

### Research aims and questions

This study examined the impact of the compounding crises that engulfed Lebanon during the COVID-19 pandemic on the schooling and healthcare opportunities and experiences of persons with disabilities. In addition, the study explored how the interaction of disability with other layers of discrimination, such as gender and socioeconomic further exacerbated the risk of exclusion from mainstream education and healthcare services, particularly for service recipients who have disabilities and low-income students with disabilities. Two research questions led this study: (a) How has the COVID-19 pandemic restricted persons with disabilities from accessing education and healthcare sectors, and (b) what are the impacts of the COVID-19 pandemic on persons with disabilities across education and healthcare sectors?

### Methods

Three qualitative methodologies were utilized to assess the impact of COVID-19 on persons with disabilities in Lebanon.

#### Documentary and literature analysis

The researchers looked at 37 existing studies and reports produced by the Lebanese Ministry of Public Health and local and international disability organizations such as IDA and OCHA. Furthermore, interviewees from UN agencies shared related rapid analysis reports and studies about the COVID-19, Beirut Port Blast, and the economic crises in Lebanon. It comprised fast analytical studies produced in Lebanon by UNICEF, ESCWA, Human Rights Watch in Lebanon and other NGOs.

#### COVID-19 awareness campaign analysis

The researchers examined and evaluated COVID-19 awareness information and short advocacy videos created by the Lebanese Ministry of Health, local and international disability NGOs, and United Nations agencies. It included reviewing their website section that provides COVID-19 digital written information, posters, infographics, and images. It also analyzed the Lebanese Ministry of Health Vaccination registration website “IMPACT.” Moreover, the analysis included reviewing five video awareness campaigns entitled “Covid safe return to school” produced by the UN Children’s Fund (UNICEF) with the collaboration of the United Nations Development Programme (UNDP) and Lebanon’s Ministry of Information. The researchers reviewed and analyzed 103 COVID-19 video awareness campaigns produced by the Ministry of Public Health (MOPH). The Lebanese Federation of the Deaf made 15 Covid-related video awareness campaigns and five videos produced by the Centre for Lebanese Studies-Disability Hub- Lebanese American University, available on their websites or Facebook pages. The review sought to determine how well the COVID-19 awareness campaigns and media material portrayed persons with disabilities in their media coverage and the degree of accessibility for persons with visual, hearing, and cognitive disabilities.

#### Interviews

Individual virtual interviews were carried out with eighteen (18) parents of students with disabilities under 18 and stakeholders from local disability groups, UN agencies, and local educational and healthcare service providers.

### Sample

The study participants were selected randomly for the virtual interviews. Eighteen service recipients and providers with different age groups, gender, and disabilities took part in virtual discussions. Three virtual interviews were held with mothers of children with disabilities who were under the age of 18. The first parent lives in Nabatiyeh, south Lebanon, and has a 10-year-old son with autism who attends a special private school. In contrast, the second interviewee is a mother who has a seven-year-old female child with Down’s Syndrome who is enrolled in a private special education school. Finally, the third mother in Bekaa has a 14-year-old autistic son who attends a school run by a UN organization. One interviewer is a male student at the age of 18, is a wheelchair user and studies at a private university. In addition, four university lecturers with and without disabilities participated in the virtual interviews. Two interviewees were male university lecturers who had visual impairments, while the remaining two were females without disabilities who had students with learning difficulties in their classes.

On the other hand, 10 stakeholders offered further education and healthcare services tailored to various disabilities were among the selected sample. This included two private special education providers for people with autism and intellectual disabilities, one educational provider for people with visual impairments, one educational provider for people with hearing disabilities, and a private higher education service provider in charge of providing digital accessibility and assistive devices for students with disabilities. On the other hand, three UN bodies provided education and healthcare services for children with physical, visual, and intellectual disabilities. The remaining three interviewees were head and school teachers who had experiences in teaching Deaf students, students with learning difficulties, ADHD, autism, and intellectual disabilities.

Ethical approval was obtained from the Institutional Review Board (IRB) at the Lebanese American University. Parents, stakeholders, and parents of students with disabilities received consent forms. The researchers explained the study thoroughly and assured the anonymity and confidentiality of all data collected.

### Data analysis

The data collected were analyzed using interpretational analysis. The recorded Zoom virtual interviews were transcribed verbatim by the researchers. The transcripts were coded using an open coding method to identify the common themes in those transcriptions. The data were compared using the constant comparative method, where the similarities and differences of the data were derived. Later, the codes were divided into five categories based on five main themes: (a) Educational and healthcare services amid COVID-19; (b) recipients’ reflections on services during the pandemic (c) Information technology accessibility provisions; (d) Emotional and mental wellbeing Services; and (e) Medical care Facilities.

On the other hand, the two researchers used an accessibility checklist to review and analyze the level of accessibility for the written COVID-19 posters and infographics and the video awareness campaigns. It included checking the written text size and font and visual contrast for people with low-visual impairment and color blindness using a complimentary color contrast checker tool named Colour Contrast Analyser (CCA). It determines the color contrast ratio of the text and the background using an eyedrop tool that complies with Web Content Accessibility Guidelines (WCAG 2.1). In addition to the visual contrast, the two researchers used the Microsoft built-in Check accessibility feature to examine whether the posters, images, and infographics had alternative descriptive text (Alt-Text) to identify the pictures that are accessible for users with visual impairments who rely on the screen reader software to access the digitalized information. On the other hand, the two researchers analyzed the COVID-19 video awareness content to identify the missing accessibility features, including sign language interpretation, closed captioning and adequate font size and color for Deaf people and those with hearing impairment; audio description for the written, visual content for Blind people and those with low vision, and simple language and graphic illustration for people with intellectual disabilities.

## Results

This part presents the living conditions and the difficulties that persons with disabilities in Lebanon faced during COVID-19, which overlapped with a multifaceted crisis in Lebanon. The researchers generated five themes that highlight the impediments that restricted persons with disabilities from gaining access to education and healthcare services in Lebanon.

### Educational and healthcare services amid COVID-19

Before the pandemic, service providers who participated in this study targeted their services to serve persons with disabilities to gain access to education and healthcare sectors; nevertheless, most of their programs could not cover the needs of service recipients across the Lebanese districts. Moreover, the economic crisis that the country has experienced since 2019 added burdens on service providers to maintain their services as they used to do before the crisis. Despite all of the challenges put on interviewed service providers during the epidemic and the economic crisis, most of them mentioned that the Lebanese Ministry of Health and the Ministry of Social Welfare did not respond to or consider the education and healthcare needs of persons with disabilities during the COVID-19 pandemic and the economic crisis. Around half of the interviewed service providers mentioned that since 2018, they did not receive financial support from the Ministry of Social Welfare (MOSA), which added financial constraints and limited them from providing educational and healthcare services for persons with disabilities.

The political uncertainty and the governmental resignations delayed the Ministry of Social Welfare (MOSA) from approving and issuing disability cards for new beneficiaries. These delays hindered most interviewed disability NGOs from providing educational and healthcare services for persons with disabilities. Furthermore, the petrol shortage and the slow internet connection disrupted online services for most interviewed service providers who tried to maintain their services during such complex and unstable conditions. In contrast, others stopped offering their services due to the October 17 uprising road closures. All interviewed service providers stated that these financial deficiencies forced them to reduce their educational and medical facilities budget. Most education service providers expressed concern about their organizations’ future viability, citing an increase in persons with disabilities and their families’ demand for social assistance. Similarly, many interviewed teachers struggled to balance their workload and devalued earnings.

The COVID-19 epidemic, and the Lebanese multi-pronged economic crisis, forced most interviewed disability organizations to change their service delivery techniques to accommodate persons with disabilities. All interviewed service providers had to shift from delivering their services face-to-face to online and used various internet platforms to provide their services. Most service providers preferred to rely on the WhatsApp application to communicate with service recipients. Half of the interviewed service providers relied on Zoom, while Microsoft Teams, Google Forms, and Live Worksheets were less used, as only 10% of service providers relied on these tools. Conversely, one education provider for the Deaf community relied on several online platforms with video calls for sign language interpretation and included accessibility features such as the generated automatic closed captioning for audible communication.

All education service providers indicated offering teacher training for using these online platforms and online resources. One of the UN agencies sponsored training courses in collaboration with a private institution. These comprised customizing COVID-19 written content to be accessible for students with sensory disabilities and facilitating communication, participation, and therapy services in an online setting. One higher education representative mentioned that their IT department developed recommendations and provided training sessions for instructors to use different online platforms and utilize available built-in accessibility features. They used voiceover over PowerPoint presentations, using Alt-text descriptive text, color contrast, text fonts and line spacing and adopted the written text for screen reader users and those with dyslexia and learning difficulties. Moreover, the training included adding open and closed captioning for audiovisual media content and adequate positioning in front of the camera for sign language interpreters and those who rely on lip-reading to access audible information.

Although many educational institutions provide instructions for students to help them utilize online platforms, not all students with disabilities manage to use them independently. One interviewer of a local disability NGO indicated that students below 10 needed parental support. In contrast, teenage students were familiar with using online platforms and PowerPoint presentations shared by their teachers during remote learning. Around half of the participants mentioned that parental engagement and follow-up were necessary to assist their younger children during online learning. One educational provider for students with autism said that her organization produced around one hundred different illustration cards that were shared on WhatsApp groups to better communicate with families and students with autism. Moreover, guidelines and instructions were given to parents to maintain the students with autism classroom routines and to minimize TV time at home since many children with autism can get irritated by certain lights, sounds, and background noises. Almost two-thirds of education service providers stated that parental help compensated for the absence of special educators whose services are most successful in person; nevertheless, they could not substitute for in-person psychosocial and other therapy services.

Recognizing that not all services could be provided online, over two-thirds of the educational service providers who participated in the study decided to return to in-school learning while adhering to safety precautions and included services for children with autism, intellectual disabilities, and Deaf students. “To allow for lip-reading parallel to safety precautions of needing to cover the mouth-nose area with masks, teachers used face shields as clear masks were not practicable and caused suffocation,” revealed a Deaf educational service provider. The students used regular face masks. On the other hand, almost a third of education service providers created a flexible plan to offer hybrid services that include both online and face-to-face instruction, forcing many teachers to work beyond their official working hours. Most interviewed service providers delivering safety and healthcare services had to arrange home visits for persons with disabilities. All INGOs distributed personal protective equipment, respiratory machines, face masks and other healthcare kits among around one hundred and twenty disability organizations.

### Recipients’ reflections on services during the pandemic

Findings from interviewing service recipients revealed that most of them could not have access to mainstream education and healthcare services during the COVID-19 pandemic and the economic crisis in Lebanon. Many relied on online services during road closures and lockdown restrictions; however, remote services were disrupted due to the shortage of gasoline and the slow internet connection. Furthermore, most of them could not attend online learning since many families could not afford to purchase tablets and PC devices for each child/student, which put stress and extra burdens on families of children with disabilities. All of the parents stated that they lived in a stressful environment. “As parents, we could not cope with the unstable political and economic situation in Lebanon, then we had to provide extra support to our children during online learning, which aggravated our stress levels and anxiety during the lockdown restrictions,” a 35-year-old mother stated.

All interviewed parents pointed out that persons with disabilities never received any support or educational aid from the Lebanese government, which does not cover the high costs of special education schooling. A mother of a 7-year-old daughter with Down syndrome mentioned that her daughter had to undergo a medical operation during the COVID-19 pandemic. One student with a physical disability said that the Ministry of Public health did not cover the costs, but rather a private insurance company as the family was not entitled to receive governmental insurance. The Ministry of Education invited several students with disabilities to learn about the barriers they encountered during the COVID-19; however, it did not take further steps or procedures to enhance online learning for persons with disabilities.

Findings from interviewing service recipients revealed that persons with disabilities in Lebanon could not gain access to mainstream education and medical care provisions during the COVID-19 pandemic. Most interviewees noted that the Lebanese government never had an inclusive and central emergency response plan nor a follow-up procedure to respond to persons with disabilities or vulnerable groups during emergencies. A 45-year-old female education provider stated, “With the absence of a centralized authority to respond to different disability groups, many local and international NGOs had to tailor their services to certain disability groups without covering all types of disabilities and populations.”

Both service providers and service recipients concluded that COVID-19 had a good and bad impact on education and healthcare services. Parents of students with disabilities pointed out that a face-to-face curriculum was more efficient and better than online learning. Most parents of students with disabilities noted that many schools reduced the curriculum content during online learning instead of focusing on educational skills knowledge, creating confusion for teachers and students with disabilities. On the other hand, interviewed education providers considered online assessments unreliable test measurements since they were uncertain whether the students managed to master the skills independently or relied on their parents to complete the given assignments and assessments. A 50-year-old female headmaster stated, “The daily face-to-face interaction with students in a classroom setting allows instructors to interact with their students and recognize their learning capabilities and limitations, which would be difficult to identify behind the screens and in an online classroom setting.”

Most schoolteachers and university lecturers talked about a decline in student and teacher interactions during online learning. A 40-year-old university male lecturer with visual impairment found out that “online learning was a different style of lecturing which was not an issue for me… what I missed in a face-to-face classroom environment is the students’ interaction and participation which was difficult to maintain in online learning settings.” Interviewed parents mentioned that their children with disabilities were less engaged with schoolmates and hardly participated with their peers during online sessions. On the other hand, a 35-year-old mother of a 9 years old boy with autism and learning difficulties noted that in a face-to-face school setting, her child could learn and interact with classmates once enrolled in grade one, more than joining his chronic age class group, which is grade four. She added, “Such interaction was not the same during a virtual classroom setting as my son could not physically interact and build a close relationship with his classmates.” Another 35-year-old female UN education provider representative added that students with and without disabilities could not apprehend nor process the given information as many got agitated and lost attention during online sessions. She said that: “Online teaching content and tools were not adapted for students with disabilities, particularly children with ADHD and those who have learning difficulties.”

Similarly, a 50-year-old female school principal echoed this by saying, “Some children with and without disabilities could not interact nor take part during online classroom environment, although they used to be more engaged in a face-to-face school setting.” Interviewed university lecturers and schoolteachers attributed the decline in academic achievement to the lack of students’ self-discipline, demotivation, and inability to focus and follow the lecturers’ instructions during virtual sessions. All parents of students with disabilities noted that their children’s attention span in class instruction was more successful as they were more attentive and could focus longer, mainly when teachers relied on visual aids and interactive teaching techniques and experiments.

On the other hand, many interviewed school teachers and lecturers were less motivated and passionate about delivering their courses online since they were unfamiliar with the new virtual working styles. More than half of them attributed the loss of “stamina” to their physical housing environment, which lacks the standard lecture or classroom design configuration. “Online learning is forcing lecturers to convert their private residential rooms into working places or classroom environments which is a problem by itself,” stated a 40-year-old university male educator with vision impairment. More than half of the interviewed lecturers were not ready to move to the new online teaching modality. According to a thirty-year-old female instructor, “online education obliged many educators to modify their teaching contents within a short period. Educators resisted altering their teaching styles as many were not ready to take that step further.” Few interviewed educators, including a 43- year-old male university lecturer, benefited from remote teaching since he did not have to drive frequently to reach his workplace and preferred to give his lectures online.

One-third of interviewed education providers and parents/students with disabilities stated parents were not always unavailable and ready to assist their children with disabilities during online learning, which hampered online learning efficiency. “As teachers, we had difficulties cooperating with parents during online learning,” said a 47-year-old female special education provider. An eighteen-year-old male undergraduate student with a disability added that parental support helps young students develop their time management skills, utilize online learning platforms, and provide a secure and safe learning virtual environment. All interviewed special education providers highlighted that Deaf students and students with cognitive disabilities, and autism spectrum, needed parental support and assistance to communicate virtually with their teachers and classmates. Moreover, many students with cognitive disabilities below the age of 18 found difficulties in operating different virtual online platforms and needed parental support; however, not all parents were able to provide that support, as many had work obligations and were not available at all times. Most interviewed parents verified the latter point where they did not have the teaching skills or the knowledge to follow up and support their children’s academic demands. Some interviewed parents attended inclusive education workshops and managed to help their children with intellectual disabilities during regular school settings and were prepared to assist them online during the pandemic. However, few parents could provide such support since they did not have the same educational knowledge and experience. A 30-year-old mother with a 7-year-old Down syndrome daughter had a flexible work schedule and coordinated with her husband to support their daughter during online learning. Both parents used various interactive educational resources to reinforce the learning concepts that their daughter took during online learning since both of them were familiar with inclusive education; however, the mother pointed out that:

Both parents and teachers have to coordinate to support students with disabilities; however, not all parents nor teachers work together on this. During the online learning, I tested out different learning techniques with my daughter, shared them with the instructors, and they put them into practice. However, as parents, we are not qualified inclusive education specialists, and we can not fill the role of shadow teachers or therapists. Schools should support our children within the remote learning model without adding extra work or tasks on parents. Coordination and collaboration between school and parents benefit students; however, giving parents the responsibility of supporting their children with disabilities in virtual learning would increase the tension between parents and children and would not necessarily work for the benefit of students with disabilities.

Likewise, a 35-year-old mother of a son who has both learning difficulties and autism added that,

As a mother who has a degree in Applied Behavioral Analysis (ABA), I knew that students with autism and learning difficulties require a variety of tools to learn. For this purpose, I developed and used different multisensory learning tools to assist my child during online learning. I dedicated my expertise and time to joining his online classes as a shadow teacher and clarifying any confusing concepts. I managed to support him because I had the experience, but what about the other parents who did not have such knowledge? I can assure you that many parents could struggle to provide such a level of support for their children with disabilities during online learning.

In other instances, parents of children with disabilities could not support their children’s needs at home because they were either overwhelmed with work and house responsibilities or could not equally provide educational support for their children with and without disabilities. According to a forty-year-old female UN education provider, some families with several children struggled to provide support and attention equally. Many gave their full attention to older children without disabilities, as some were in their final school years, resulting in less attention to children with disabilities’ learning demands.

Whilst all interviewed education providers had to shift to online learning, less than a quarter of them knew about the digital accessibility features in the platforms they used. One female IT accessibility specialist in her forties stated that the private higher education institution had already adopted digital accessibility before COVID-19. Still, the academic staff did not receive any training workshops to enhance their teaching methods to be aligned with the accessibility provisions. One 30-year-old female instructor at a higher education program for individuals with disabilities added, “Our school team relied on several online learning platforms during the October 17 Road closures, but not all teachers were aware of the different accessibility features.” About half of the interviewed education providers concluded that preserving and improving the knowledge and usage of technology tools could enhance the interaction between students and educators, improving online learning quality. A 45-year-old female principal of a special centre for students with autism recommended that online services be kept as an option for any upcoming unstable events, weather circumstances, or student illnesses that may prohibit students from attending face-to-face classes.

More than half of the interviewed parents of students with disabilities and education providers stated that many students with disabilities did not benefit from online learning. They claimed that students with ADHD, severe autism, and sensory disabilities did not benefit from online learning as they needed several interactive learning and communication methods. A 50-year-old male representative for an NGO providing inclusive education for students with visual impairments noted that many schools had to reduce online the curriculum. Another 50-year-old female teacher at a school for the Deaf pointed out that online learning necessitated relying on the Arabic language as a universal language for ease of communication, which was difficult to understand for students using sign language. The teacher stated, “Learning sign language via online setting was quite difficult, and most learners failed to apprehend it. Although sign language is similar to any foreign language as it has its grammar and idioms, the signs used are attributed to different community cultural backgrounds which are more difficult to teach via the online setting.”

On the other hand, less than one-third of the interviewees noted that online learning was effective for certain persons with disabilities. They stated that students with physical disabilities found online education more accessible than their physical classroom settings. Moreover, some students with mild to moderate autism benefited from online learning as it allowed them to learn at their own pace and have their privacy to learn as they “were happier when isolated from class hustles.” Another benefit of online learning was “independency and self-learning,” as claimed by a 50-year-old male local disability NGO representative who said,

A face-to-face classroom typically requires an hour of explanation to ensure that all students grasp a particular concept; in an online environment, however, you are constrained by the zoom duration. Therefore, rather than only depending on the teacher, the students will need to be autonomous and attempt to obtain answers afterwards through classmates, other family members, web page resources, or YouTube.

### Information technology accessibility provisions

Most interviewed parents of students with disabilities and education and healthcare service providers experienced difficulties in IT technology which was inaccessible for many disability types. Most of them were unfamiliar with the accessibility features provided by some online communication platforms. Around one-quarter of the interviewed education providers who serve children with autism and visual impairment used different multisensory tools to stimulate sensory processing during class settings. Other education providers use electronic devices such as iPad and touch tablets to meet various student learning demands, particularly for students with limited motor skills and who cannot use pen and paper for notetaking. Hence electronic devices such as iPads and computers were used as an alternative method for note-taking strategies. In addition, visual resources such as videos, digital boards, YouTube videos, and PowerPoint representations were used for students who rely on visual aids to learn.

“Most conferencing tools have built-in accessible provisions; however, not many of us know about such features before COVID-19. We need to activate such features to enhance access to online meetings” said a female IT accessibility specialist in her mid-forties who works in a private higher education institution. Another 50-year-old female interviewee who works as a teacher in a school for the Deaf revealed that their school used “Free and simple social platforms and programs such as WhatsApp, TikTok, Google Maps, and YouTube. We also chose Zoom since it has screen-sharing provisions, cameras, and a built-in chat for question-and-answer sessions. We also utilized simple PowerPoint presentations with simple text and proper spacing.”

As teaching approaches changed throughout COVID-19, the demand for using assistive aids for students with disabilities expanded. Around one-third of those interviewed depended on particular accessibility features to accommodate their students with disabilities. A 50-year-old male university instructor with visual impairment utilized screen readers software products such as JAWS and MVDA. Another female special education teacher in her forties used synchronous Smart Boards during online screen-sharing courses and gave out touch screen computers to pupils with intellectual disabilities. “There are particular applications for speech therapists and automatic verbal transcription such as speech-to-text and text-to-speech features that we utilized throughout online learning,” she continued.

Most interviewees noted a lack of financial resources to provide assistive technology. They emphasized that IT accessibility service was already so expensive that few persons with impairments and their families could afford it. However, due to Lebanon’s economic crisis, most of them could not acquire or maintain them. Students with visual disabilities found difficulties in purchasing Braille letters on Lebanese laptop keyboards. Many interviewees who are blind and have visual impairments noted that screen readers could only read pdf files in English or French, but they cannot read Arabic pdf files. They also stated that most academic sites featuring photos, graphs, and diagrams are unavailable to screen readers since they do not include alternate text descriptions.

On the other hand, a female teacher at a Deaf school stated that sign language was unsuitable for online learning platforms. Many of the automated closed captioning are not 100% accurate nor synchronized within verbal communication.

### Emotional and mental wellbeing services

Most interviewees experienced anxiety, fear, and uncertainty during the lockdown restrictions with the increased number of COVID-19 deaths and hospitalized cases, in addition to having relatives and friends departing the country in response to Lebanon’s unstable political and economic circumstances. Most interviewed parents of children with disabilities were overwhelmed and emotionally strained due to their workload and their children’s educational demands. Most parents stated that their children with disabilities lacked the motivation to study and learn online. A 50-year-old female teacher in a school for the Deaf noted that most of her students were “exhausted and distracted.” Another 40-year-old female respondent from an international disability group stated that students with learning disabilities and those with epilepsy experienced anxiety due to online learning obligation overload.

Similarly, special education providers for children with intellectual disabilities stated that their students felt lonely and dissatisfied with online learning and that some missed their campus, friends, and professors. On the other hand, children with cognitive disabilities became hyperactive and restless during the lockdown restrictions. A female parent of a child with autism added that “My son missed joining extracurricular and sports activities that he used to have before the lockdown restrictions.”

Most interviewed education providers offered their students mental health and wellbeing online sessions. Some used WhatsApp chats to check on their students. In contrast, others encouraged group interaction by engaging them in various activities to express their feelings and communicate their problems using social platforms such as Zoom. Some education providers, on the other hand, provided mental health assistance and activities for teachers and parents of children with disabilities since teachers and caregivers, too, were drained emotionally during the lockdown restrictions. Autism awareness short video clips regarding routine and autism outbursts were also distributed to parents of children to ease stress and anxiety among families of children with disabilities.

Furthermore, several interviewed instructors participated in online group sessions offered by their school counselors to share their feelings and relieve stress. Only a few respondents stated that virtual mental health sessions were ineffective and could not provide the same privacy and confidentially they used to have during in-person sessions. Other educational providers had to cancel academic obligations, postpone homework deadlines, and give extra time for students to complete their assignments to support the wellbeing of instructors, parents, and students with and without disabilities.

### Medical care facilities

Interviews with persons with disabilities and their parents in Lebanon indicated that many of them could not receive healthcare services before the COVID-19 outbreak. Many said the Lebanese government failed to acknowledge their medical requirements throughout the epidemic. Most parents could not take their children with disabilities for routine medical checkups during the lockdown restrictions, and others feared catching the virus in healthcare institutions and hospitals. One mother could not take her son with autism to a doctor during lockdown restrictions. “His health condition worsened and demanded surgery … if we were able to take him immediately to a doctor, we could have avoided the surgery,” added his mother. All interviewed parents of children with disabilities could not find any relevant information on the Ministry of Health website about the number of persons with disabilities who were caught or died due to the virus. Interviewed healthcare providers stated that such information was communicated internally and individually inside disability groups. Moreover, interviewed parents of children with disabilities pointed out that the Ministry of Health should add persons with disabilities to its data collection. Such information should be available to all citizens to prevent the spread of the virus among persons with and without disabilities. On the other hand, the Lebanese Ministry of Public Health (MOPH) developed 103 movies and animated awareness campaigns regarding COVID-19, prevention of COVID-19, quarantine, self-isolation, health and wellbeing, and COVID-19 vaccines to increase awareness about COVID-19 pandemic. Whilst the posters and animated videos provided safety measures to prevent the spread of the virus among children, adults, and the elderly, persons with disabilities were not included among the target population. On the other hand, reviewing and analyzing the short films indicated that most videos were inaccessible to many persons with disabilities. Only six of the 103 videos, or 5% of the total, featured Lebanese Sign Language interpretation. However, none of the videos included close captioning/subtitles. Many videos used carton illustrations and animation to communicate key safety measure instructions; however, most of the visual content lacked voiceover and audio description, two important accessibility features for Blind people and those with visual impairments. Findings from evaluating the level of accessibility at the Ministry of Public Health (MOPH) website and COVID-19 posters, and “IMPACT” platform revealed that both websites did not meet the accessibility provisions for people with visual impairments, those with limited language and IT literacy, and old generation. One Deaf education provider stated that most Lebanese live news, mainstreaming announcements and daily updates regarding COVID-19 did not include sign language interpretation. The United Nations International Children’s Emergency Fund in Lebanon (UNICEF) website posted several COVID-19 safety instructions and advocacy campaigns about the COVID vaccine information. Most of the written posters, images and graphics of the UNICEF web page in Lebanon lacked descriptive alternative text descriptions (Alt-text). They did not meet the WCAG 2.0 visual contrast ratio of at least 4.5:1, making it inaccessible for Blind people and those who rely on screen readers to access digital information. While some of the UNICEF COVID – Safe Return to School video announcements used sign language and embedded English subtitles, most did not have closed Arabic captioning. Most interviewees encountered difficulties accessing governmental COVID-19 audiovisual, and digital content as much of this information did not meet the accessibility provisions for people with sensory disabilities. Furthermore, the Lebanese Federation of the Deaf created COVID-19 videos with Sign Language interpretation; however, most lacked closed captioning. On the other hand, the Centre for Lebanese Studies and its Disability Hub platform at the Lebanese American University produced several COVID-19 awareness campaigns accessible to people with visual, hearing, and intellectual impairments. The Arabic and English versions included visual animation, audio description for the visual content and open and closed captioning. All interviewed people with visual impairments stated that most of the governmental COVID-19 awareness video campaigns relied on written material without voiceover, which hindered them from accessing the video content. According to one female instructor who works in a school for the Deaf, most videos lack sign language interpretation. They are inaccessible to Deaf people and those who rely on closed captioning. Many interviewees with sensory disabilities were upset and frustrated as they could not gain access to audiovisual and digital media content before and during the pandemic. A 50-year-old male university instructor with a visual impairment said, It frustrates me when I can not access important information, TV program announcements and advocacy campaigns that do not have voiceover. It is our right to have accessible digital and audiovisual media content to learn more about events and information around us. Most interviewees stated that the Lebanese government relies on television and social media platforms to convey official and important announcements and awareness videos inaccessible to people with sensory and intellectual disabilities. With the lack of government-accessible information on the COVID-19 epidemic, many interviewed family members of children with disabilities monitored their children to ensure that they followed the COVID-19 hygiene routines to prevent them from getting infected. A female instructor at a Deaf school mentioned that several organizations volunteered to improve the accessibility of various governmental campaigns. The Lebanese Federation of the Deaf (LFD) collaborated with the Ministry of Communication to produce several COVID-19 awareness campaigns and uploaded many of them on their Facebook page. Similarly, the Centre for Lebanese Studies at the Lebanese American University posted on its Disability Hub platform several COVID-19 awareness campaigns that show some of the daily life experiences of persons with disabilities during the lockdown restrictions. Findings from reviewing these videos revealed that the Lebanese Federation of the Deaf (LFD) videos had sign language interpretation accessible for the Deaf community; however, they are not accessible to people with visual impairments. On the other hand, the COVID-19 videos produced by the Centre for Lebanese Studies were fully accessible, as they contained an audio description of the visual content and subtitles in both English and Arabic languages.

When asked about their satisfaction with the accessibility provisions for the COVID-19 vaccination platform named “IMPACT,” most interviewed parents of children with disabilities and educators with disabilities encountered barriers when they wanted to fill out the online application form. Many of them could not document their disability types as the online application did not list disability as one of the variable options. To overcome such limitations, many chose the following classifications “other,” “diseases,” and “chronic illness,” which led to associating disability with illnesses and the medical model of disability. A 30-year-old female NGO representative mentioned that many persons with disabilities were entitled to receive the vaccine as they were among the second-stage vaccine priority groups; however, their names could not be registered under the disability category on the “IMPACT” platform.

Most interviewees concluded that the priority for persons with disabilities to take the vaccine should have been irrespective of people’s disabilities. They noted that vaccination prioritization should be based on people’s medical condition and age regardless of disability, in addition to people’s dependence on caregiver support. Vaccination should be given to first-liners and caregivers. One male interviewee with visual impairment commented that the Lebanese government outlooked persons with disabilities in receiving the vaccine while deploying its COVID-19 vaccination plan.

As for the Vaccination platform, “Impact,” around half of the interviewees accessed the platform to register their names. They mentioned that the platform was inaccessible to people with specific types of disabilities and older generations who were unfamiliar with completing online registration forms. They concluded persons with visual impairment, intellectual disabilities, and those not accustomed to digital processes would need assistance to complete the registration. A 50-year-old male local disability organization interviewee supporting children with visual impairment stated that the “IMPACT” platform IT developers and designers did not involve persons with disabilities during the design phase.

On the other hand, the Ministry of Health held two vaccination marathons set up to vaccinate people with and without disabilities enrolled on the “IMPACT” platform on a walk-in basis. Most participants noted that the COVID-19 marathons are more suitable for people without disabilities. Two special education providers mentioned that their students with intellectual disabilities and those with autism did not participate in the vaccination marathon as most could not cope with being in big crowds. Hence many parents of children with intellectual disabilities did not attend these Marathon vaccinations. On the other hand, some children with autism who participated in the first Marathon rejected another trip to the vaccination station since many had negative experiences at the centre during their first shot.

One of the UN organizations requested home immunization services, but their request was rejected. Other private disability NGOs contacted COVID-19 committee members to vaccinate children with disabilities in their organization, but both requests were denied. On the other hand, persons with disabilities were required to show their disability card to receive the vaccine; however, not all of them were registered with the Ministry of Social Affairs and could not obtain the disability cards. To overcome such limitations, the Ministry of Health replaced the disability card with a medical report for persons with disabilities who did not register their disability within the Ministry of Social Affairs. However, not many families of children with disabilities managed to obtain medical reports, as many could not afford to pay the costs of such reports, nor they had the time to follow up on such documentation within a short period. Most parents of students with disabilities and education providers with disabilities stated that not all the allocated vaccination centres were accessible for different disability types. A female teacher for the Deaf said that accessibility measures were limited to mobility impairments; however, people with sensory and intellectual disabilities did not have any accessibility provisions that meet their particular needs, resulting in low participation during the vaccination Marathon.

## Discussion

This section summarizes the main findings from interviews with 18 parents of children with disabilities and healthcare and education service providers. Although the sample is small, it shows the barriers that persons with disabilities in Lebanon encountered during COVID-19 and can give insight to the Lebanese government and policymakers so they can enhance their services to become more inclusive. Although the sample size is small, it demonstrates the challenges that individuals with disabilities in Lebanon faced during COVID-19 and can provide insight to the Lebanese government and policymakers so that they can improve their services to be more inclusive in the future. The analysis of the services before and during the COVID-19 epidemic demonstrated that the pandemic restricted service access for persons with and without disabilities. Yet, persons with disabilities faced additional barriers that hindered them from accessing online learning and medical care services during the lockdown restrictions. Five key themes highlight the obstacles within the educational and medical care services that the Lebanese government and policymakers should address to respond to the needs of persons with disabilities during the Covid-19 pandemic or any upcoming emergency.

### The impact of multifaceted crisis in Lebanon

The outcome finding of the study demonstrates the significant destabilizing crisis influence of Lebanon’s multifaceted crisis, which is occurring concurrently with the pandemic and service providers and persons with disabilities. Many hospitals and schools in Beirut were partially or fully demolished by the August 4th Port Blast in 2019 (35), while schools and universities had to close for several weeks due to October 17, 2019, uprising before COVID-19 lockdown restrictions (20). As a result, education and medical care services became out of reach for persons with disabilities during the lockdown restrictions. Furthermore, Lebanon’s economic crisis put the education and healthcare sector in a critical condition. Many service providers faced the possibility of ceasing their services due to the financial shortfalls caused by the country’s economic failure, in which expenses grew, but funding resources remained constant. The financial crisis has also impacted students with and without disabilities. Many could not have proper access to online education due to the interrupted and slow internet connection caused by the country’s gasoline shortage and the financial constraints that hindered many persons with disabilities from purchasing electronic devices and tablets.

The COVID-19 lockdown restrictions and the unstable political and economic situation in Lebanon affected the mental health of service providers and students with disabilities, who felt stressed, anxious, depressed, and uncertain of their future. Persons with disabilities had other emotional distress; those with cognitive disabilities were disoriented, lonely and powerless. Children with autism became more hyperactive as they could not cope with such situations. Service providers, students with disabilities and their parents attended several remote and online mental health and counseling sessions; the most favored in-person sessions as they provided a safe and secure environment that online sessions lack.

### Absence of emergency preparedness inclusive plan

Lebanon’s education and medical care sectors did not consider the needs of persons with disabilities and vulnerable groups during the multifold crisis and the COVID-19 pandemic. The absence of an emergency preparedness inclusive plan attributed to excluding persons with disabilities from gaining access to public services before the COVID-19 pandemic (20). The Lebanese government enforced the closure of schools, special educational organizations and centres on February 29, 2020, to limit the spread of the COVID-19 virus (20). The lockdown restrictions obligated education service providers to rely on remote learning to compensate for missed and interrupted academic programs. Although many education providers adopted online learning settings, findings showed its ineffectiveness for Deaf learners, students with intellectual disabilities and those with autism, who preferred to join in-school learning while adhering to safety procedures.

The study revealed that the Lebanese government outlooked the educational and medical care needs of persons with disabilities during the COVID-19 pandemic. Although the Ministry of Social Welfare had obligations to provide financial support for disability organizations and institutions, findings revealed that they did not receive such funds since 2018. This finding is confirmed in a rapid analysis report produced by Human Rights Watch, where several special education schools were owed significant sums of funds by the government (20). On the other hand, the Lebanese Ministry of Education and the Ministry of Public Health did not provide financial aid to support persons with disabilities who could not afford to cover any urgent medical costs or educational assistive devices. The Lebanese Ministry of Health provided statistical data about the infected cases and death tolls but did not provide such data about persons with disabilities. Providing statistical data about the Lebanese population, including persons with disabilities who caught or died from the virus, can assist the government in tracking the infected cases and controlling the virus’s spread among people with and without disabilities. Findings revealed that providing accessible medical information and awareness campaigns about COVID-19 for all its citizens could have helped the government prevent the virus’s spread among people with and without disabilities.

### Limited access to regular medical checkup services

Persons with disabilities in Lebanon could not have access to medical care services during the pandemic since hospitals were overcrowded with patients infected by the virus (20, 37) in addition to the limited accessibility provisions in Lebanese hospitals (34), many interviewed parents could not take their children with disabilities to regular medical follow-up visits due to the COVID-19 lockdown restrictions and the possibility of catching COVID-19 in healthcare centres and hospitals, putting a few at a higher risk of having severe health conditions. To avoid such risks, parents adhered to the COVID-19 hygiene protocols to prevent their children from catching the virus. Other parents sought assistance from disability organizations to obtain medications and respirators for their family members with disabilities who have chronic health conditions (20).

### Lack of accessible COVID-19 awareness information

COVID-19 hygiene awareness information is essential for preventing the spread of the virus among persons with disabilities who rely on mobility devices and auxiliary aids to carry on with their daily activities. Others use their hands to touch surfaces to navigate their surroundings (28), and many depend on caregivers and personal assistance and can be at a higher risk of catching the virus (16). Findings from reviewing and analyzing COVID-19 awareness information revealed that most of the written and audiovisual information did not meet the accessibility provisions for people with visual, hearing impairment, and intellectual disabilities, nor were they accessible for the older generation to have little literacy knowledge.

Many disability-led organizations stepped forward to fill the lack of accessible information by producing and disseminating several COVID-19 awareness campaigns and information accessible for the Deaf and people with visual and intellectual disabilities.

The Lebanese government created a National COVID-19 Vaccine panel and organized a National COVID-19 Deployment and Vaccination Plan in preparation for vaccine deployment that will be distributed equally among Lebanese citizens and people residing in Lebanon (38). Yet, findings from interviews with disability organizations revealed that the COVID-19 panel lacked a disability representative, and the vaccination plan did not have any procedures to deploy the vaccination for persons with disabilities (39). Furthermore, findings revealed that the “IMPACT” vaccine registration platform did not have “disability” as one of the population categories. This limited users to document their disabilities under the “diseases” or “chronic illnesses” category, reinforcing and supporting the medical model of disability by associating disability with medical illness. Furthermore, the “IMPACT” platform application did not meet the accessibility provisions for people with visual impairments who rely on screen readers, while people with intellectual disabilities and those unfamiliar with digital procedures needed family assistance to complete the registration form. While the vaccination plan permits education service providers to register their teachers and staff to receive the vaccines, findings revealed that the registration procedures did not include students with and without disabilities who could have received the vaccines through their schools and disability organizations.

To speed up the vaccination deployment, the COVID Vaccination panel organized Vaccination Marathon that aimed to operate vaccination on a walk-in basis for any citizens registered on the “IMPACT” platform; however, it outlooked persons with disabilities. Findings revealed that persons with disabilities entitled to take the vaccine encountered physical barriers since most of the designated vaccination units and centres lacked accessibility provisions. To compensate for that limitation, the Ministry of Health coordinated with the Ministry of Social Welfare and organized a Pfizer Vaccination Marathon for persons with disabilities, which was operated in March 2021. Many interviewed disability organizations were considered short notice and needed better coordination. Although many disability-led groups advocated for the designated Marathon for persons with disabilities by producing and disseminating information about the Marathon in an accessible format for people with sensory and intellectual disabilities, not many persons with disabilities managed to participate in such an event. Findings from interviews with disability organizations and families of children with disabilities revealed that many persons with disabilities did not have disability cards. In contrast, others could not afford to pay the costs of obtaining medical documentation about their disabilities which hampered them from receiving the vaccination.

### Lack of preparation for inclusive remote education

Most interviewed education providers concluded that remote learning did not support the different learning demands of students with and without disabilities. Education providers used various learning tools and resources inaccessible to Deaf students and students with visual and intellectual disabilities. Teachers noted that online evaluations and assessments were unreliable as they could not determine their students’ ability to acquire specific skills and knowledge in such virtual settings. Both students and teachers experienced a lack of motivation in remote learning settings since they could not interact and participate in discussions as they used to have in-person classroom environments. It resulted in a drop in student performance levels for children with and without disabilities and average and below-average students. Most students with disabilities in Lebanon do not enroll in mainstream education since many public schools do not provide designated programs that meet their educational demands and needs (20). Findings revealed students with sensory and cognitive disabilities, students with ADHD and those who rely on shadow instructors were the most who did not benefit from online learning since schools reduced the curriculum rather than focusing on necessary acquired skills and knowledge.

Moreover, many used tools do not have accessibility built-in features, and while some have such features, not many teachers are aware of their existence, and others do not know how to use them. In addition, many teachers were not ready to learn new teaching methods and tools despite the training workshops offered during the online school preparations. On the other hand, many parents were not prepared and did not have time to assist their children with disabilities during online learning classes, particularly parents who had children with intellectual disabilities who required more attention and support. Another reason that hampered students with disabilities from benefiting from online learning was the lack of assistive learning devices, the inability of some parents to purchase such learning devices, and the lack of governmental financial support to cover the costs of providing tablets and laptops (20). Findings revealed few students benefit from online learning, including students with mobility impairments, students with mild autism, and students who manage to focus and perform better when removed from the in-person classroom environment.

## Conclusion, implications, and limitations

### Conclusion

This study highlighted that persons with disabilities in Lebanon were disadvantaged, isolated, and excluded from having access to public education and medical care facilities before the pandemic; however, they experienced extra barriers and isolation during the COVID-19 pandemic. Both the Lebanese Ministry of Education and the Ministry of Public Health completely ignored the rights of persons with disabilities to gain access to these two essential services during the pandemic. The government must draft inclusive procedures and policies with the collaboration and involvement of disability representatives and organizations to ensure that persons with disabilities demands are met in any upcoming emergency event or situation. It entails a better understanding and implementation of the Lebanese Law 220/2000 and the UN Convention on the Rights of Persons with Disabilities (CRPD). The Lebanese Ministry of Education has to analyze the education demands of persons with disabilities and the financial costs of purchasing assistive devices. It must develop an inclusive and accessible curriculum that matches the diverse learning styles and students with and without disabilities, which is based on acquiring skills and knowledge rather than condensing the intake contents. In addition, the schools should provide more psychological support, assistive devices and different multisensory learning tools/techniques to cater to diverse student learning demands.

Furthermore, inclusive workshop training must be provided to all levels of personnel. Coordinators and administrators must be willing to help and support instructors through the transition. The Ministry of Education should lead on licensing for inclusive education schools, which must meet the inclusive education policies and procedures that meet the different learning and educational demands of students with and without disabilities. On the other hand, the Ministry of Public Health should improve its services to meet the diverse requirements of persons with disabilities. Accessibility measures should be planned ahead of time as part of a long-term strategy for inclusion so that people with different disability types can access clinics, hospitals, and any related medical information provided on its website.

Furthermore, inclusive training must be delivered to all the medical team and staff so they can address the various requirements of patients with disabilities. Hospitals and medical centres should enhance their physical environment to become accessible for different disabilities. Sign language interpreters must be among the staff teams to facilitate communication between Deaf patients and medical doctors. Similarly, personal assistants/caregivers should be allowed to accompany persons with disabilities whenever needed. Finally, the priority for COVID-19 vaccination for persons with disabilities must be based on the medical condition and age rather than disability.

### Implications and recommendations

In Lebanon, the government should take proactive measures to draft and implement inclusive policies in academic and medical care sectors to serve all its populations, including persons with disabilities. It is advised that a comprehensive, inclusive strategy is drafted with the collaboration and consultations of persons with disabilities, educational experts and medical specialists to ensure that it covers all measures and procedures in any upcoming emergencies.

The primary step would entail a better understanding of the Lebanese Law 220/200 and the UN Convention on the Rights of Persons with Disabilities (CRPD) would lead to a better implementation of these Laws, mainly because the Lebanese parliament is in its process of ratifying the UN Convention. It requires revising Law 220/200 to align with the CRPD articles and notions. Moreover, developing inclusive service criteria is essential to ensure that all disability needs are acknowledged once providing a particular service. Moreover, creating a long-term vision for inclusion that includes accessibility measures and provisions implemented in any upcoming emergency events and monitoring and evaluating the implementation of accessibility measures/standards is vital in enhancing the services over time. The second suggestion focuses on educational services. Policymakers should adopt an inclusive action plan to ensure that students with disabilities gain access to educational services in person or via online learning. It includes thoroughly assessing the financial cost of persons with disabilities’ education needs during lockdown situations, revising curricula for students with disabilities based on the required skills and knowledge rather than quantity reduction, and providing other multisensory learning tools/techniques, psychological support, and assistive devices and reasonable accommodations for different disability types. The third recommendation targets academic staff and teams who should take inclusive training workshops to enhance their teaching skills and accommodate the diverse needs of students with and without disabilities.

Furthermore, providing an open resource platform that includes different teacher training resources and guidelines on conducting studies and writing reports about disability-related topics. These extra training courses and resources could benefit schools that aim to obtain official inclusive accreditation. The fourth and last recommendation is to prioritize vaccination for persons with disabilities regardless of disability but, instead, take into account their medical condition and age.

### Research challenges and limitations

Researchers encountered many constraints while conducting the study about the impact of COVID-19 on persons with disabilities in Lebanon during the lockdown restrictions.

The researchers could not conduct interviews with instructors, university faculty members, and university students due to the time needed to gain IRB permission, which took about 4 months (March 26–June 25). However, during this time, the researchers reviewed and analyzed several studies and reports and compiled a list of possible volunteers who fall within the study’s parameters. In 1 month, 18 people participated in the study, including parents and students with disabilities, local and international disability NGOs, teachers and lecturers, and medical care service providers. Although the researchers sent several emails to government representatives, they did not receive emails from them. The researchers made Follow-up telephone calls as part of the research invitation procedures; however, many of them could not have time to participate in virtual interviews.

Due to power outages and slow internet connections, many participants declined to participate in this study. Moreover, the researchers were unable to complete several virtual interviews. They had to reschedule other meetings due to the interrupted internet connection and the frequent electrical cut-off, which also interrupted the transcription and note-taking activities. To compensate for such constraints, researchers used WhatsApp calls to complete the interviews, while some participants sent audio notes after their virtual interviews were disrupted.

## Data availability statement

The raw data supporting the conclusions of this article will be made available by the authors, without undue reservation.

## Ethics statement

The studies involving human participants were reviewed and approved by the Lebanese American University. The patients/participants provided their written informed consent to participate in this study.

## Author contributions

All authors listed have made a substantial, direct, and intellectual contribution to the work and approved it for publication.

## Conflict of interest

The authors declare that the research was conducted in the absence of any commercial or financial relationships that could be construed as a potential conflict of interest.

## Publisher’s note

All claims expressed in this article are solely those of the authors and do not necessarily represent those of their affiliated organizations, or those of the publisher, the editors and the reviewers. Any product that may be evaluated in this article, or claim that may be made by its manufacturer, is not guaranteed or endorsed by the publisher.
